# Motivational climate of group exercise sessions in nursing homes

**DOI:** 10.1186/s13690-020-00425-y

**Published:** 2020-05-12

**Authors:** Alexia Charles, Fanny Buckinx, Alexandre Mouton, Jean-Yves Reginster, Olivier Bruyère

**Affiliations:** 1 Department of Public Health, Epidemiology and Health Economics, WHO Collaborating Center for Public Health Aspects of Musculoskeletal Health and Ageing, Division of Public Health, Epidemiology and Health Economics, University of Liège, CHU-Sart-Tilman, B23, Quartier Hôpital, Avenue Hippocrate, 13, 4000 Liège, Belgium; 2 Department of Sport and Rehabilitation Sciences, University of Liège, Liège, Belgium; 3 Chair for Biomarkers of Chronic Diseases, Biochemistry Department, College of Science, King Saud University, Riyadh, KSA Saudi Arabia

**Keywords:** Motivational climate, Exercise group, Physical activity, Older people, Institutionalization

## Abstract

**Background:**

Motivational climate in exercise group environments would have an impact on adherence, effort and enjoyment. We examined the motivational climate among nursing home residents who were involved in group exercise sessions.

**Methods:**

This cross-sectional study was conducted in 10 nursing homes of Liège area that offer group exercise sessions. Sociodemographic data (age, sex, body mass index), cognitive status (by the Mini Mental State Examination) and independence in activities of daily living (by the Katz Scale) were retrieved in the medical records. The “Abbreviated-Perceived Motivational Climate in Exercise Questionnaire” was translated into French and then administered face to face with a clinical researcher. This is composed of 6 ego-involving climate items (corresponding to rivalry, comparison and favoritism) and 6 task-involving climate items (corresponding to valorization, individual efforts, self-improvement and cooperation). Each item is ranged on a 5-point Likert scale ranging from 1 (not at all focused on ego or task) to 5 (totally focused on ego or task). Each subscale has a total score expressed as an average.

**Results:**

A total of 102 subjects of exercise group sessions were included (84.3 ± 7.7 years and 83 (81.4%) women). The mean score of task-involving and ego-evolving motivational climate was respectively 3.57 (SD = 0.67) and 1.52 (SD = 0.49), suggesting that the motivational climate was more focused on the task-involving climate than on ego-involving climate. Some items results were of particular interest: 55.9% of the respondents found that the instructor doesn’t remark/reward when they try hard, 63.7% said that the instructor doesn’t encourage mutual aid and 38.2% found that instructor doesn’t encourage to do new exercises.

**Conclusions:**

Participants tended to perceive motivational climate as more task-involving than ego-involving. The absence of individual positive feedback, new exercises and mutual aid were also highlighted.

## Background

There is a growing body of literature that recognizes the importance of physical activity in older people. It has previously been observed that engagement in physical activity has a positive impact on functional abilities [1, 2], mobility [3], cognitive status [4, 5], quality of life [6, 7], risk of falls [8] and mortality [9, 10]. In addition, the International Association of Gerontology and Geriatrics (IAGG) and the World Health Organization (WHO) have emphasized the importance of physical exercise in the quality of nursing home care [11].

As we showed in a previous study, these sessions lead to additional physical activity (and thus higher energy expenditure) over a week [12]. Moreover, physical activity programmes for older individuals appear to maintain physical activity levels and physical quality of life [13]. However, the level of physical activity of nursing home residents is generally low [14], and it seems important to motivate them to move more. Therefore, special consideration should be given to group exercise sessions offered in nursing homes [15, 16].

Some studies emphasize the importance of creating positive and pleasurable interactions and a supportive environment, which would encourage residents to participate in organized activities more regularly [17, 18]. The motivation of the residents as well as the presence of a monitor ​​seem to play a role in participation and adherence to soft gym sessions in nursing homes [19, 20]. These observations converge in the motivational climate of the group exercise sessions proposed in nursing homes. It has been suggested that a motivational climate, defined as “a psychological environment guiding the goals and motivations of an individual” [21], has a favourable effect on physical performance, psychological state and commitment to exercise [22–26]. However, enjoyment is also related to the motivational climate generated by the instructor and his or her communication style [24]. Given the lack of information concerning this topic in nursing homes, we aimed to explore the motivational climate of group exercise sessions in nursing homes by interviewing participants using a validated questionnaire and to evaluate the association between the motivational climate and the characteristics of subjects.

## Methods

### Design and sample

This cross-sectional study included residents of ten nursing homes in the Liège area who attended group exercise sessions on a regular basis (i.e., at least once a week for at least 1 month) and were willing to collaborate on this research project. The research was conducted between July 2019 and September 2019. The subjects were included if they were aged 65 years or older, voluntarily participated in the study, had approved the informed consent form and were involved in the group gym session. The exclusion criteria were cognitive impairment (a mini mental state examination [MMSE] score ≤ 21 out of 30 [27]) and a native language other than French. The study protocol was approved by the Ethics Committee of the University Teaching Hospital of Liège under number 2019/159.

### Subjects’ characteristics

Participants’ sociodemographic and clinical data were collected from residents’ medical records. The data considered were age, sex, body mass index (BMI), walking aid, independence in activities of daily living assessed by Katz’s scale [28] and cognitive status evaluated by the MMSE [29]).

### Motivational climate

The French version of the “Abbreviated Perceived Motivational Climate in Exercise Questionnaire (PMCEQ-A)” was administered face-to-face to each participant by a clinical researcher [30]. Beforehand, the initial designers of the questionnaire, which had been developed in English, agreed to have the questionnaire translated into French. The questionnaire was translated by our division according to the guidelines of Beaton et al. [31]. This questionnaire is used to assess the motivational context perceived by the subjects during group exercise sessions. It is composed of 12 items, including 6 items related to the task-involving climate and 6 items related to the ego-involving climate. The task-involving climate is focused in particular on the cooperation among participants, personal improvement or the valorisation of everyone. On the other hand, the ego-involving climate is characterized by the feeling of rivalry between the participants, the embarrassment of not knowing how to carry out the exercise or the feeling that only the best are valued. Each item is coded with a scale ranging from 1 (strongly disagree) to 5 (strongly agree). Each subscale has a total score expressed as an average (i.e., from 1 to 5). A higher score reflects a higher ego-involving climate or task-involving climate.

### Statistical methods

The collected data were processed using SPSS software version 25 (IBM Corporation, Armonk, NY, USA). First, a descriptive analysis of the different variables was performed. The normality of these variables was tested using the Shapiro-Wilk test and QQ-plot. Normally distributed continuous quantitative variables were expressed as the mean and standard deviation (SD), while those with abnormal distribution were expressed as the median and percentiles (25th Percentile and 75th Percentile). The qualitative variables were described in terms of number and frequency (%). Then, the association between subjects’ characteristics and motivational climate was evaluated with linear regressions (with adjustment on age and sex).

## Results

### Study population

Among 124 subjects meeting the inclusion criteria, a total of 102 subjects were included in this study (18 refused to participate). Table 1 presents the characteristics of the sample. The mean age of the participants was 84.3 ± 7.7 years, and 81.4% were female. The mean scores of task-involving and ego-evolving motivational climate were 3.57 (SD = 0.67) and 1.52 (SD = 0.49), respectively.

**Table 1:** Characteristics of study population (*N* = 102)

**Variables**	**Mean ± SD or Median (P25-P75) or N (%)**
**Age (years)**	84.3 ± 7.7
**Sex (women)**	83 (81.4)
**BMI (kg/m**^**2**^**)**	26.3 ± 5.4
**Walking aid**	
**No**	27 (26.5)
**yes**	75 (73.5)
**MMSE score (/30)**	25.3 ± 3.3
**Katz score (/32)**	11 (9–13)
**Motivational climate**	
**Ego-involving (1–5)**	1.5 ± 0.5
**Task-involving (1–5)**	3.6 ± 0.7

*SD* Standard deviation, *P25* 25th Percentile, *P75* 75th Percentile, *N* Number; *BMI* Body mass index, *MMSE* Mini-Mental State Examination

### Motivational climate

The response rates of 12 items of the A-PMCEQ are reported in Table 2. When looking separately at each ego-involving climate item, the results showed that 89.2% of respondents thought that residents were not hesitant to ask the instructor for help, 92.2% thought that they did not feel embarrassed if they did not know how to use the equipment or perform an exercise, 93.1% related that they are not encouraged to do better than others, 79.4% said that they are not excited when they do better than others, 92.2% said that the instructor paid attention to all of the participants and 97.1% said that the instructor does not make it clear who he/she thinks are the most fit.

**Table 2:** Results for ego-involving and task-involving climate items in Abbreviated Perceived Motivational Climate in Sport Questionnaire (*N* = 102)

	**Items**	**Completely agree N(%)**	**Partly agree** **N (%)**	**Neutra**l **N(%)**	**Partly disagree N(%)**	**Completely disagree N(%)**
**Ego-involving**	**Members are hesitant/embarrassed to ask the instructor/staff for help**	2 (2.0)	8 (7.8)	1 (1.0)	21 (20.6)	70 (68.6)
	**The instructor/ staff gives most of his/her attention to only a few members (high status, most fit, etc.)**	1 (1.0)	7 (6.9)	0 (0)	13 (12.7)	81 (79.4)
	**Members feel embarrassed if they do not know how to use the equipment or perform the exercise/skill/drill**	0 (0)	9 (8.8)	0 (0)	26 (25.5)	67 (65.7)
	**Members are encouraged to do better than other members**	5 (4.9)	0 (0)	2 (2.0)	28 (27.5)	67 (65.7)
	**The instructor/staff makes it clear who he/she thinks are the most fit and/or skilled members**	0 (0)	3 (3.0)	0 (0)	17 (16.7)	82 (80.4)
	**Members are excited when they do better than their fellow classmates**	1 (1.0)	20 (19.6)	0 (0)	23 (22.5)	58 (56.9)
**Task-involving**	**The instructor/staff encourages us to try new exercises/skills**	33 (32.4)	28 (27.5)	1 (1.0)	25 (24.5)	14 (13.7)
	**Members of all fitness levels are made to feel valued**	49 (48.0)	46 (45.1)	1 (1.0)	4 (3.9)	2 (2.0)
	**Members are rewarded and noticed when they try hard**	20 (19.6)	22 (21.6)	3 (3.0)	34 (33.3)	23 (22.5)
	**The instructor/staff encourages members to help each other**	12 (11.8)	24 (23.5)	1 (1.0)	17 (16.7)	48 (47.1)
	**The instructor/staff emphasizes always trying your best**	67 (65.7)	26 (25.5)	0 (0)	6 (5.9)	3 (2.9)
	**The focus is to keep improving on each exercise/skill each class session**	43 (42.)	35 (34.3)	0 (0)	22 (21.6)	2 (2.0)

*N* Number, *%* Percentage

Regarding the items related to the task-involving climate, 93.1% of respondents found that residents of all fitness levels are made to feel valued, 76.5% of respondents said that the focus is to keep improving on each exercise/skill during each class session, 91.2% thought that the instructor emphasizes always trying one’s best, 55.9% found that the instructor does not remark/reward when they try hard, 63.7% found that the instructor does not encourage participants to help each other and 38.2% found that the instructor does not encourage participants do new exercises.

### Motivational climate and characteristics

Our secondary objective was to evaluate the association between participants’ characteristics and perceived motivational climate. Our goal was to assess whether some characteristics (i.e., age, sex, BMI, cognitive status and Katz score) could have an influence on the perceived ego-involving or task-evolving climate. The results in Table 3 show that only independence was negatively associated with ego-involving climate (β = − 0,049, 95% CI = 0,020-0,078, *p* = 0,001). This means that residents with more dependency reported higher levels of ego-involving climate. There was no association between the other characteristics and motivational climate (ego-involving or task-evolving climate).

**Table 3:** Association between characteristics and ego-evolving climate/ task-evolving climate (*N* = 102)

	**Ego-involving climate**					**Task-involving climate**				
**Variables**	**Estimate**	**Standard Error (SE)**	**95% CI**		***p*value**	**Estimate**	**Standard Error (SE)**	**95% CI**		***p*value**
**Age**	−0.003	0.006	−0.016	0.009	0.613	−0.009	0.009	−0.027	0.008	0.293
**Sex (women)**	−0.170	0.123	− 0.413	0.074	0.170	−0.018	0.172	−0.360	0.323	0.916
**BMI**	−0.012	0,010	−0.032	0.007	0.218	0.013	0.014	−0.014	0.041	0.337
**Katz score**	0.049	0,015	0.020	0.078	**0.001**	−0.005	0.020	−0.045	0.034	0.789
**MMSE score**	−0.008	0.015	−0.038	0.022	0.592	−0.007	0.021	−0.049	0.035	0.744

*SE* Standard error, *C*I Confidence interval, *BMI* Body mass index, *MMSE* Mini-Mental State Examination

## Discussion

This present research examined the motivational climate of group exercise sessions held in nursing homes. Our findings seem to suggest that the nursing home residents perceived their motivational climate as more task-oriented than ego-oriented. This trend is consistent with reports by other authors who have studied younger populations. Indeed, several studies recommended creating task-involving motivational climates in sport and physical education settings rather than ego-involving climates [32–35]. Interestingly, the task-involving climate has been positively related to the intrinsic motivation of individuals (i.e. interest, perception of competence, and effort-importance) [36]. In contrast, the negative influence of the ego-involving climate on satisfaction and intrinsic motivation has been frequently noted [37, 38].

The detailed analysis of A-PMCEQ showed interesting results. Regarding ego-involving climate items, the majority of subjects (i.e., over 78% for each item) do not experience rivalry, favouritism or concerns about mistakes. Similarly, the caring climate usually established in nursing homes should negatively relate to perceptions of an ego-involving motivational climate [39]. On the other hand, regarding task-oriented climate items, a large number of subjects experienced feelings of being valued, of individual improvement and of being encouraged to do their best. However, three item results were of particular interest. First, more than half of the respondents found that the instructor did not remark/reward when they tried hard. Nevertheless, previous studies have shown that positive feedback provided by instructors helped participants to gauge their personal progress and was an additional motivator [40–42]. Moreover, rewarding the effort of participants is likely to generate satisfaction and pride [24]. Second, almost two-thirds of the subjects said that the instructor did not encourage them to help each other. One possible explanation is that the instructors do not encourage this behaviour because they want to guarantee the safety of participants. However, it has been reported that social interactions with other resident-participants can serve as physical activity motivators [43]. Third, one-third of participants found that the instructor did not encourage them to try new exercises. Nonetheless, other studies have confirmed that nursing home residents need variety and innovation in their exercise programmes [43, 44].

When we investigated the association between characteristics and motivational climate, we found a relationship between the perceived ego-involving motivational climate and the dependency of the participants. This means that those who were more dependent on help in their activities of daily living perceived a more ego-oriented motivational climate than those who were more independent in their activities of daily living. This would suggest that they felt they were being compared with others during group exercise sessions. There is some evidence to suggest that adapted exercises seemed to be essential for older people [40, 45]. Therefore, several variants of the exercises could be proposed to the residents in order to elicit an optimal execution level in each resident, thus leading to a higher motivational level. Moreover, communication strategy by the exercise group instructor could also be important to address the functional capacity of those with poorer function and an interesting way to attempt to change motivational climate [46].

Regarding the practical implications of our results, it seems important to implement an adequate motivational climate during exercise sessions. In the literature, some recommendations for quality physical education interventions were established with, for example, the PAMIA principles [47]. Pleasure, achievement, movement, interaction and autonomy are the 5 principles of these recommendations. Although they are not specific to older people, these can be related to the elements identified in our study and are the key elements to prioritize when implementing group exercise sessions in nursing homes. The 3 elements highlighted in our study are related to the interaction among participants (for mutual aid), achievement (for diversified exercises and adapted exercise with a sufficient level of difficulty) and movement (for positive feedback) [47]. These elements are also mentioned in the theory of self-determination as well as in the three psychological needs of Bandura (1977), namely, competence (adapted exercises and positive feedback), autonomy (diversified exercises and choices), and relatedness [48, 49].

It is important to recognize the possible bias and limitations of this study. The cross-sectional nature of this study allows us to generate descriptive results regarding motivational climate but no causal inferences between variables. The generalisability of these results is subject to certain limitation because our analysis was based on a relatively small sample. Additionally, the composition of the sample of oriented nursing home residents exclusively leads to a bias in the representativeness of the population. The results should be interpreted with caution because the A-PMCEQ was developed for the general population and not specifically for older people. Moreover, the personality and enjoyment of the nursing home residents have not been explored in this study although they could play an important role in the perception of the motivational climate.

## Conclusion

In conclusion, the A-PMCEQ is an interesting tool and highlighted a climate in nursing homes that is especially task-oriented. On the other hand, it does not add value to the ego-task distinction. Moreover, the lack of individual positive feedback, new exercises and mutual aid were emphasized. Special attention to adapted exercises should also be required. Future research is recommended to further investigate the quality of the intervention at the task level rather than the ego-task distinction.

## Data Availability

The datasets used and analysed during the current study are available from the corresponding author on reasonable request.

## Abbreviations

IAGG: International Association of Gerontology and Geriatrics
WHO: World Health Organization
MMSE: Mini mental state examination
BMI: Body mass index
PMCEQ-A: Abbreviated Perceived Motivational Climate in Exercise Questionnaire
SD: Standard deviation
